# Application of atopic dermatitis control tool (ADCT) for daily practice in a community-based dermatology clinic

**DOI:** 10.3389/fmed.2026.1790489

**Published:** 2026-04-01

**Authors:** Toshiya Ebata, Yuko Hayakawa-Ventimiglia, Qin Lin

**Affiliations:** Chitofuna Dermatology Clinic, Tokyo, Japan

**Keywords:** ADCT, atopic dermatitis, daily practice, IGA, long-term control

## Abstract

Atopic dermatitis (AD) is a chronic inflammatory skin disease with a relapsing and remitting course. It is important to maintain remission over the long term in order to preserve patients' quality of life and achieve treatment goals. To identify the status of long-term control, the application of patient-reported outcome measures (PROMs) is useful. We applied the Atopic Dermatitis Control Tool (ADCT) to our everyday practice. ADCT is a holistic scale with 6-items covering multiple aspects of disease control. Between July and December 2021, all the patients with AD over the age of 12 years who visited our clinic were evaluated using the ADCT and Investigator's Global Assessment (IGA) on each visit. A total of 238 patients (mean age 34.4+/−14.5 years old; 63% male) completed the ADCT. In total, 568 sets of measurements were obtained. The average ADCT score was 7.8+/−4.9, and the ADCT score significantly correlated with that of the IGA. 62.9% of patients were categorized as having good control (ADCT score < 7) at least once during the study period. The ADCT score of those who regularly visited was 6.5+/−3.9 (*n* = 68), while the irregular patients had a score of 10.0+/−4.9 (*n* = 75). The ADCT score of new patients on their first visit was 11.1+/−5.7 (*n* = 29), and upon remission, the score was 3.9+/−2.9. Based on our experience, measuring ADCT in all AD patients is feasible. By continuing this practice over the long term, it is expected to understand patients' long-term disease control status and support treatment decision-making.

## Introduction

In the management of Atopic Dermatitis (AD), it is important not only to induce remission but also to maintain a state of remission over the long term in order to preserve patients' quality of life (QOL) and achieve treatment goals (1, 2). Atopic Dermatitis Control Tool (ADCT) is a recently developed self-administered questionnaire designed to assess long term disease control of AD from the patient's perspective (3). It consists of six items addressing AD symptoms (eczema and severe itching) and the impact of AD (bothersomeness, sleep, daily activities, mood/emotions).

Unlike other tools that evaluate solely symptom severity such as the Eczema Area and Severity Index (EASI) and the Patient-Oriented Eczema Measure (POEM), and the quality of life such as Dermatology Life Quality Index (DLQI) the ADCT serves a different purpose which is long-term control. There remain numerous unknowns regarding its usefulness and application in both clinical trials and routine clinical practice. In this study, we report our experience using the ADCT in daily practice at a community -based dermatology clinic.

## Subjects and methods

Subjects were AD patients aged 12 years and older who visited the Chitofuna Dermatology Clinic between July and December in 2021. From July to October, the ADCT was used on a trial basis with selected patients. From November onward, the ADCT was administered at every visit for all patients.

ADCT was measured using the Japanese version of ADCT (www.adcontroltool.com). The initial measurement was conducted after the consultation, where patients who agreed to use the ADCT filled it out in the waiting room. Results were recorded in their medical charts. From the second visit onward, patients completed the ADCT prior to the consultation, and the results were referenced during the consultation.

For data analysis, medical chart information was retrospectively collected to assess the following: (1) distribution, mean, median, and mode of total ADCT scores, (2) ADCT scores in relation to validated Investigator's Global Assessment (vIGA) severity (4), (3) number of cases achieving a total score of 6 or less that indicates good disease control, (4) scores according to visit pattern and treatment method, (5) comparison with POEM scores. The validation study of the ADCT reported that an ADCT score ≥7 indicates that atopic dermatitis is not adequately controlled. Accordingly, an ADCT score ≤ 6 was considered to represent well-controlled disease (3). Visit patterns were predetermined based on clinical practice. Regular attendees were defined as patients who routinely scheduled their next appointment at the time of consultation and attended follow-up visits at regular intervals of approximately 1–2 months without missing appointments. Irregular attendees were defined as patients who visited the clinic only when symptoms worsened or when their medications ran out. We also evaluated how incorporating the ADCT influenced treatment selection and communication with patients, as well as potential issues in its use.

For statistical analysis, differences in ADCT scores based on vIGA severity and visit pattern were tested using the Kruskal–Wallis *H*–test and Mann–Whitney *U*-test with Bonferroni correction. To address potential within-patient clustering due to repeated measurements, a sensitivity analysis was performed using one observation per patient (the first visit during the study period). The correlation between POEM and ADCT scores was assessed using Spearman's correlation coefficient. A *p*-value of < 0.05 was considered statistically significant. Statistical analyses were performed using Microsoft Excel 2019 with ystat (ver8). This study was approved by the Ethics Committee of Noguchi Dermatology Clinic (No. 30, 2021.12.21).

## Results

**Figure 1:**
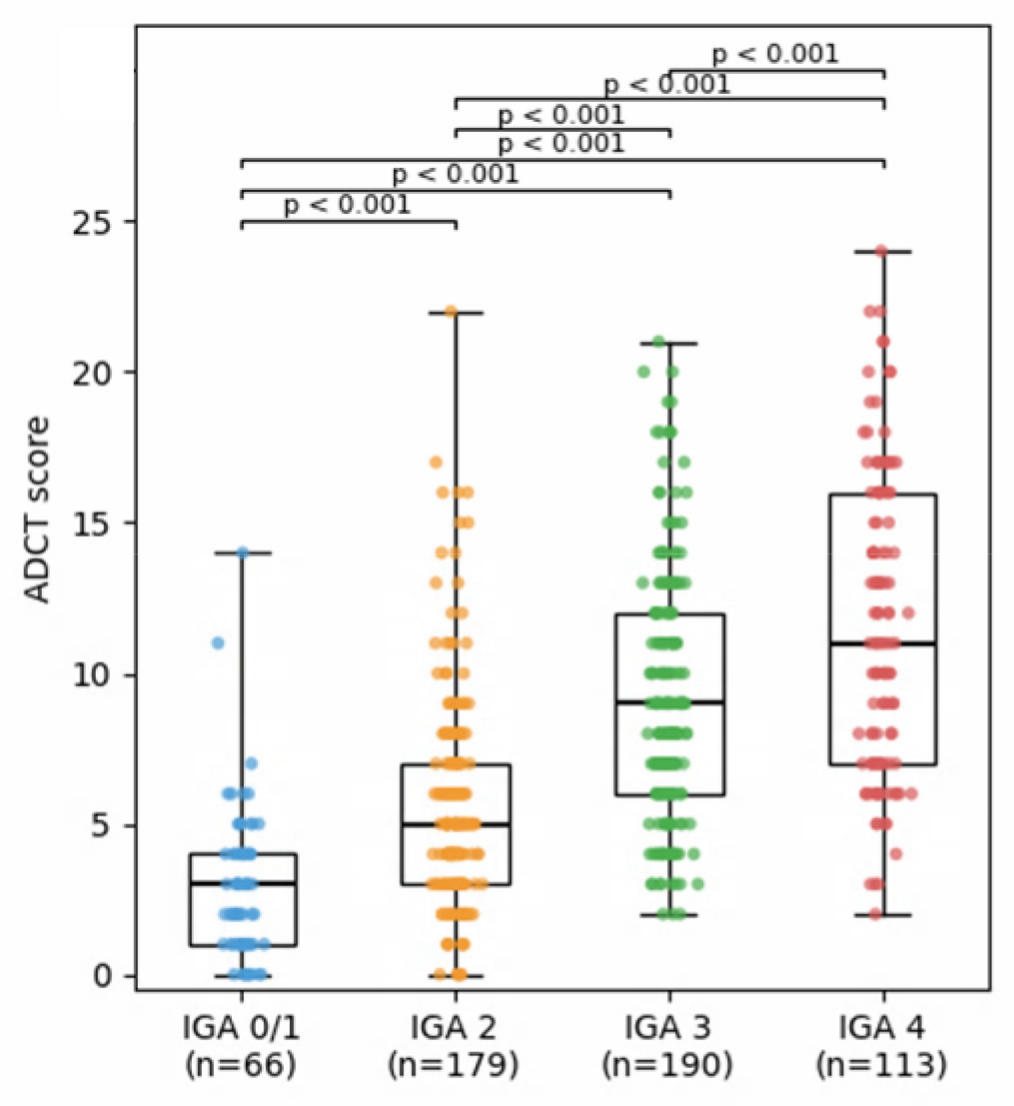
Distribution of Atopic Dermatitis Control Tool (ADCT) scores according to validated Investigator's Global Assessment (vIGA) severity categories. ADCT scores increased with higher IGA severity levels. Data are shown as box-and-whisker plots (median and interquartile range), with individual observations overlaid. Differences among groups were assessed using the Kruskal–Wallis test followed by pairwise Mann–Whitney *U* tests with Bonferroni correction. All pairwise comparisons between IGA categories were statistically significant (*p* < 0.001).

A total of 238 patients (150 males, 88 females) completed the ADCT. The mean age was 34.4 ± 14.5 years (range: 12–72). Distribution of vIGA scores at initial ADCT assessment was: vIGA 0/1-−19 patients (8.0%), vIGA 2-−75 (31.5%), vIGA 3-−86 (36.1%), vIGA 4-−50 (21.0%), and vIGA not recorded-−8 (3.4%). Some patients provided multiple responses during the 6-month study period, with a total of 568 responses. After excluding 7 with missing data, 561 were included in the analysis. Mean number of visits was 2.4 per patient with maximum of 7 visits, observed in two patients. The average ADCT score was 7.8 ± 4.9; the median was 7, and the mode was 4. A strong correlation was observed between ADCT scores and vIGA-assessed disease severity (Figure 1). Results were similar in a sensitivity analysis restricted to the first visit per patient. The POEM was also assessed simultaneously with the ADCT on 244 occasions, showing a significant correlation (correlation coefficient: 0.79). Of the 238 patients with complete data, 148 (62.2%) achieved an ADCT score of 6 or less at least once, indicating good disease control. Table 1 shows ADCT scores according to visit pattern and treatment method. Regular attendees had lower scores compared to irregular attendees and new patients. In patients, especially new ones, who successfully achieved remission induction, low ADCT scores were observed at that time. Remission was standardized across patients and determined clinically when inflammatory lesions had resolved and patients no longer reported severe pruritus with sleep disturbance, based on agreement between the physician and the patient. Among those treated with dupilumab, the majority achieved and maintained scores of 6 or less.

**Table 1:** ADCT scores according to visit pattern and treatment status.

Group	ADCT score, median (IQR)	Patients (*n*)	Responses (*n*)	*P* value
Visit pattern				
Regular attendees	6 (4–9)	68	290	
Irregular attendees	9.5 (6–13)	75	132	<0.001
New patients	11 (6–15)	29	29	<0.001
Treatment status				
Remission induced	3 (2–6)	32	37	–
Dupilumab	4 (2–6)	9	19	–

Values are presented as median (interquartile range). Statistical comparisons were performed only among visit-pattern groups using the Kruskal–Wallis test followed by Mann–Whitney *U* tests with Bonferroni correction. Treatment-status categories are shown descriptively.

## Discussion

In the management of AD, the importance of maintaining good disease control over a long period is self-evident but is particularly challenging (2, 5). The international research initiative Harmonizing Outcome Measures for Eczema (HOME), which aims to establish global standards for evaluating symptoms and treatment outcomes in AD clinical trials, recommends the repeated use of validated tools adopted by HOME such as EASI, POEM, itch Numerical Rating Scale (NRS), and DLQI to assess disease control, including both objective signs and patient-reported outcomes (1). Furthermore, two comprehensive tools for evaluating long-term control from the patient's perspective, ADCT and Recap of Atopic Eczema Control (RECAP) have been newly developed and were endorsed at the 7th HOME meeting (6).

At our clinic, we introduced the ADCT and found it highly practical. Patients could complete it in just 2–3 min during the waiting time before their consultation. We were able to score the responses before the consultation began, gaining a general overview of the patient's condition over the past week. We then recorded the total and itemized scores in the medical chart, which helped guide the clinical encounter. Recognizing its utility, we decided to implement the ADCT routinely for all patients starting in November 2021.

Other Patient-Reported Outcome Measures (PROMs) used in AD include the itch NRS, POEM, and DLQI. These tools show high inter-correlations with ADCT (correlation coefficients around 0.6–0.8) (7, 8), yet the ADCT along with RECAP stands out for its ability to quickly provide a comprehensive understanding not only of symptoms but also of the broader impact of AD in a single brief questionnaire (9). While significant correlations have been observed between ADCT scores and the severity of skin symptoms, some cases show discrepancies: high ADCT scores despite mild skin symptoms, or low scores despite more severe findings. The former may reflect patients in the itch-dominant group, characterized by severe pruritus despite mild dermatitis, or may suggest a high subjective disease burden and potential psychosomatic factors. In contrast, the latter may occur in patients who, due to chronic disease, have become accustomed to severe symptoms or have decreased motivation for treatment. Therefore, physician-based monitoring of skin lesions over time remains important. While ideally EASI should be measured in detail, it is often impractical in busy clinical settings. Thus, at our clinic, we opted to use the vIGA at each visit for practical assessment.

The favorable ADCT scores observed in the group that attended medical appointments regularly and consistently can be reasonably interpreted as an expected outcome. Nevertheless, the ability to quantitatively demonstrate a difference in ADCT scores between this group and those with irregular visits is considered meaningful, particularly from the standpoint of patient education and adherence promotion.

Each item in the ADCT has established thresholds that indicate good disease control, and beyond just the total score, elevated scores in specific areas—such as itch, sleep disturbance, or impact on daily life and emotions—can serve as cues to further explore the patient's disease burden and promote communication with the patients. In some cases, where ADCT scores fluctuated significantly over a 6-month period, the data helped guide the decision to initiate biologic therapy. This underscores the ADCT's utility in treatment decision-making as well.

Limitations of this study include its single-center nature, the relatively small number of cases, and the short observation period of 6 months. The results may differ from those obtained at university hospitals or regional general hospitals with different patient populations. Moreover, for the evaluation of long-term disease control, a longer longitudinal study is necessary. However, the primary aim of this study was to assess the feasibility of routinely measuring ADCT in daily clinical practice. Our results demonstrate that such routine assessment is feasible. Continued use of ADCT in routine practice over longer periods will allow future evaluation of long-term disease control. Although ADCT scores were strongly correlated with vIGA, discrepancies between the two measures were observed in some patients and warrant further investigation.

While ADCT has gained attention in the literature for evaluating treatment effects in severe cases (10) and predicting disease progression after discontinuation of biologics (11), its utility is not limited to such selected patient populations. The distinguishing feature of this study is that ADCT was assessed at every visit for all AD patients seen at the facility, thereby exploring the feasibility and potential benefits of incorporating ADCT into routine clinical practice. Based on our experience, measuring and recording ADCT routinely in adult AD patients is feasible and continued use of this tool may help clinicians better understand patients' disease control over time and support treatment decision-making in daily practice.

## Data Availability

The raw data supporting the conclusions of this article will be made available by the authors, without undue reservation.
